# Circulating dividing plasma cells with giant dumbbell‐shaped nuclei

**DOI:** 10.1002/jha2.330

**Published:** 2021-10-28

**Authors:** Elisa Lin, Prasad Koduru, Mingyi Chen

**Affiliations:** ^1^ Medical School, UT Southwestern Medical Center University of Texas System Dallas Texas USA; ^2^ Department of Pathology, UT Southwestern Medical Center–Biocenter University of Texas System Dallas Texas USA

**Keywords:** clinical, multiple myeloma, myeloma

A 62‐year‐old male with a history of Immunoglobulin G kappa multiple myeloma diagnosed in May 2021 was treated with three cycles of RVD [lenalidomide, bortezomib, and dexamethasone]. He was admitted in August 2021 for increasing M spike of light chains and lactate dehydrogenase, as well as worsening anemia and thrombocytopenia. The peripheral blood smear showed increased rouleaux formation and occasional bi‐nucleated giant atypical plasma cells with dividing dumbbell‐shaped nuclei (3%) (panel A, 100x objective), concerning for evolving plasma cell leukemia. The bone marrow aspirate and core biopsy revealed sheets (∼60%) of plasmacytic infiltrates (panel B, 40x objective), including the aforementioned atypical plasma cells. Immunophenotyping by flow cytometry on the bone marrow aspirate and peripheral blood confirmed kappa‐restricted monotypic plasma cells expressing bright CD38, CD56, and CD138. Cytogenetic and fluorescence in situ hybridization analysis demonstrated t(4;14) and loss of *TP53*. Next‐generation sequencing confirmed an Immunoglobulin heavy‐chain translocation with t(4;14)(p16.3;q32.2). The patient was diagnosed with refractory plasma cell myeloma with circulating plasma cells and was further treated with the KD‐PACE [carfilzomib, dexamethasone, thalidomide, cisplatin, doxorubicin, cyclophosphamide, and etoposide] protocol despite the persistence of the disease.

**FIGURE 1:**
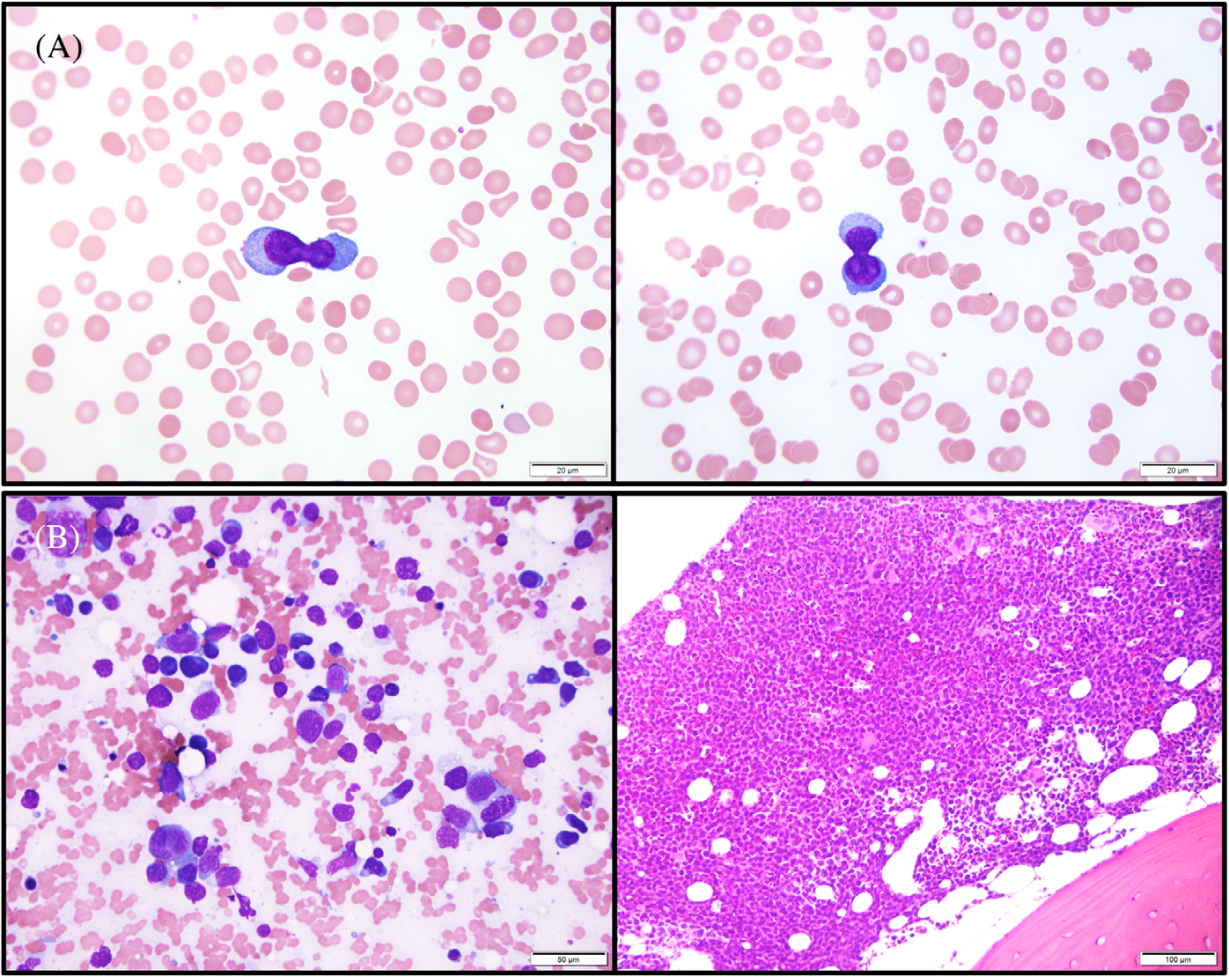
Upper panel A, peripheral blood smear shows atypical plasma cells with giant dumbbell‐shaped dividing nuclei. Low pane B, bone marrow smear and core biopsy show sheets of plasma cells infiltrate in the marrow space.

This case is remarkable because plasma cells are terminally differentiated and usually do not divide. The unusual observation seen here may represent the penultimate stage of mitosis. The disseminated dividing neoplastic plasma cells in the peripheral blood may reflect more aggressive tumor biology with a poor prognosis.

